# The Effectiveness of Virtual Reality in Improving Balance and Gait in People with Parkinson’s Disease: A Systematic Review

**DOI:** 10.3390/s25154795

**Published:** 2025-08-04

**Authors:** Sofia Fernandes, Bruna Oliveira, Sofia Sacadura, Cristina Rakasi, Isabel Furtado, João Paulo Figueiredo, Rui Soles Gonçalves, Anabela Correia Martins

**Affiliations:** 1 Coimbra Health School, Polytechnic University of Coimbra, Rua 5 de Outubro, 3045-043 Coimbra, Portugal; a2022145733@estesc.ipc.pt (S.F.); a2022115072@estesc.ipc.pt (B.O.); a2022137208@estesc.ipc.pt (S.S.); a2022134158@estesc.ipc.pt (C.R.); a2022147978@estesc.ipc.pt (I.F.); jpfigueiredo@estesc.ipc.pt (J.P.F.); ruigoncalves@estesc.ipc.pt (R.S.G.); 2 H&TR-Health & Technology Research Center, Coimbra Health School, Polytechnic University of Coimbra, Rua 5 de Outubro, 3045-043 Coimbra, Portugal; 3 Center for Rehabilitation Research, Health School, Polytechnic of Porto, Rua Dr. António Bernardino de Almeida, 4200-072 Porto, Portugal

**Keywords:** virtual reality, Parkinson’s disease, physiotherapy, balance, gait

## Abstract

Background: Virtual reality (VR), often used with motion sensors, provides interactive tools for physiotherapy aimed at enhancing motor functions. This systematic review examined the effects of VR-based interventions, alone or combined with conventional physiotherapy (PT), on balance and gait in individuals with Parkinson’s disease (PD). Methods: Following PRISMA guidelines, eight randomized controlled trials (RCTs) published between January 2019 and April 2025 were included. Interventions lasted between 5 and 12 weeks and were grouped as VR alone or VR combined with PT. Methodological quality was assessed using the PEDro Scale. Results: Of the 31 comparisons for balance and gait, 30 were favored by the experimental group, with 12 reaching statistical significance. Secondary outcomes (function, cognition, and quality of life) showed mixed results, with 6 comparisons favoring the experimental group (3 statistically significant) and 4 favoring the control group (1 statistically significant). Overall, the studies showed fair to good quality and a moderate risk of bias. Conclusions: VR-based interventions, particularly when combined with PT, show promise for improving balance and gait in PD. However, the evidence is limited by the small number of studies, heterogeneity of protocols, and methodological constraints. More rigorous, long-term trials are needed to clarify their therapeutic potential.

## 1. Introduction

Parkinson’s disease (PD) is among the fastest-growing neurological disorders globally [1], with an estimated incidence of approximately 14 cases per 100,000 inhabitants annually, rising significantly with age [2]. PD presents with a wide range of symptoms, including non-motor manifestations such as cognitive decline and depression. Motor symptoms, particularly bradykinesia, tremors, rigidity, balance impairments and gait disturbances, are especially prominent and have a significant impact on functional mobility [3].

Among motor functions, balance and gait are particularly compromised, severely limiting activities of daily living and quality of life, largely due to the heightened risk and incidence of falls [4,5]. Falls are, in fact, a major global health concern, frequently resulting in fractures, head injuries, and, in severe cases, mortality [4].

Consequently, rehabilitation targeting balance and gait is of paramount importance for individuals with PD. Physiotherapy plays a central role in mitigating the motor impairments associated with the condition [5].

In recent years, Virtual Reality (VR) has been increasingly explored as a rehabilitation tool for individuals with PD, particularly for improving balance and gait, both in clinical and home-based settings. Its growth is partly driven by its integration with emerging technologies such as artificial intelligence, telerehabilitation, and wearable sensors for movement analysis [6,7]. These systems vary widely in terms of immersion level, interaction modality, and feedback type.

VR systems deliver multisensory feedback by combining visual, auditory, and sometimes tactile stimuli. This sensory input can bypass impaired basal ganglia pathways and engage alternative neural circuits reaching the premotor cortex and supplementary motor area. Such a mechanism may compensate for the lack of internal cues in PD, thereby facilitating improved motor control [8].

Through sensors embedded in headsets or external controllers, VR may help alleviate motor symptoms such as impaired balance, freezing of gait and altered cadence. This is achieved by stimulating movement and weight shifting either in immersive (body-driven) or non-immersive (avatar-based) environments [6]. Additionally, wearable motion sensors, due to their small size, lightweight design, and low energy consumption, have proven useful in both clinical settings and real-world activity monitoring. These devices provide data on walking speed, step length, cadence, and physical activity patterns, enabling evaluation of patient function across settings [9].

In this context, the present review builds upon the 2019 systematic review “Effects of virtual reality rehabilitation training on gait and balance in patients with Parkinson’s disease” by Lei et al. [10] which served as a foundational assessment of the field. Its objective is to critically evaluate recent randomized controlled trials (RCTs) examining the efficacy of VR-based interventions, either applied solely or in combination with conventional physiotherapy, on balance and gait outcomes in individuals with PD. The comparison is made primarily against conventional physiotherapy alone, in line with the defined PICOS framework.

## 2. Materials and Methods

### 2.1. Data Sources and Search Strategy

This systematic review followed the PRISMA 2020 guidelines (Preferred Reporting Items for Systematic Reviews and Meta-Analyses) and employed EndNote Basic (Online), software for reference management. The full PRISMA checklist is available in Tables S1 and S2, Supplementary Materials. A comprehensive search was conducted across three electronic databases: PubMed, Web of Science, and PEDro, including all records available up to 23 April 2025. The search strategy included the following terms and their synonyms: “Parkinson’s Disease”, “Physical Therapy Modalities”, “Exercise Therapy”, “Virtual Reality”, “Postural Balance”, “Postural Control”, “Gait”, “Walking”, and related synonyms.

The review was prospectively registered in the PROSPERO database (CRD420251106766) to ensure transparency and reproducibility [11].

The search strategies were as follows: for PubMed, a combination of MeSH terms and title/abstract keywords was used; for Web of Science, terms were searched using the TS field; and for PEDro, the search term “Parkinson’s Disease AND Virtual Reality” was applied to titles and abstracts. The full queries for PubMed and Web of Science are detailed in Appendix A.

No date or language restrictions were applied during the initial database search; however, a filter was subsequently applied in EndNote to include only RCTs published from 1 January 2019, onwards.

### 2.2. Research Question and Study Selection

The research question was formulated according to the PICOS framework (Population, Intervention, Comparison, Outcomes, and Study design). Studies involving individuals clinically diagnosed with PD at Hoehn and Yahr stage 4 or below were included, regardless of age, gender, or duration of illness. The intervention consisted of physiotherapy incorporating VR technology, either as a standalone intervention or combined with conventional physiotherapy, which included exercise and motor rehabilitation. VR was defined as rehabilitation training delivered via computer-generated simulations, where users engage in real-time interactions within a virtual environment providing multisensory input. The comparison group received conventional physiotherapy alone. Primary outcomes focused on balance and gait. Only RCTs were eligible; studies employing other research designs were excluded.

Study selection was independently performed by two pairs of reviewers, each independently screening titles and abstracts to determine eligibility. Discrepancies were resolved through consultation with a third reviewer. No artificial intelligence tools or semi-automated screening platforms were used. All articles included were written in languages understood by the reviewers; hence, no translation tools were necessary.

Only interventions involving interactive motion-sensing VR systems were considered. No restrictions were placed on the frequency or duration of the VR-based rehabilitation. All VR and conventional physiotherapy sessions were supervised by qualified physiotherapists to ensure participant safety and treatment fidelity.

### 2.3. Types of Outcome Measures

The primary outcomes included measures related to balance and gait. In the domain of balance, several validated instruments reported in the included studies were considered, such as the Berg Balance Scale (BBS) [12,13,14,15], which assesses static and dynamic balance, and the Mini-BESTest (Mini Balance Evaluation Systems Test) [16], which evaluates multiple domains of postural control. The Limit of Stability (LOS) [16] was also taken into account, as a measure of an individual’s ability to shift their center of mass without losing balance. In addition, balance confidence was considered through the Activities-specific Balance Confidence Scale (ABCS) [15,16] and the Falls Efficacy Scale-International (FES-I) [14,17], the latter specifically addressing fear of falling. Regarding gait, the outcome measures considered included the 10-Meter Walk Test (10MWT) [14,18,19], used to assess walking speed, as well as gait speed measured over alternative distances. The 6-Minute Walking Test (6MWT) [14] was included to evaluate walking endurance. Functional gait was considered through the Dynamic Gait Index (DGI) [13] and the Functional Gait Assessment (FGA) [12,19]. Additionally, the Timed Up and Go Test (TUGT) [12,14,16,18,19] was considered a widely used measure for assessing fall risk. More global measures were also included, such as the Performance Oriented Mobility Assessment (POMA) [14,17], which comprises items related to both gait and balance, and the third part of the Unified Parkinson’s Disease Rating Scale (UPDRS III) [12,14,15,16].

Secondary outcomes encompassed multiple domains including function, cognition, and quality of life. Functional outcomes were evaluated through the Five Times Sit-to-Stand Test (5xSTS) [16], which assesses lower limb strength and transfer capacity, as well as the Barthel Index (BI) [17] and the Functional Independence Measure (FIM) [14], both used to assess independence in activities of daily living. Additional measures incorporated the Disabilities of the Arm, Shoulder and Hand Questionnaire (DASH) [13] and the second part of the Unified Parkinson’s Disease Rating Scale (UPDRS II) [15]. Cognitive function was measured by the Montreal Cognitive Assessment (MoCA) [19]. Quality of life was assessed using the Parkinson’s Disease Questionnaire (PDQ-39) [16], along with the generic health status surveys SF-12 [17] and SF-36 [13].

### 2.4. Study Selection and Data Extraction

The titles and abstracts of the identified records were initially screened by four independent reviewers, working in pairs. Duplicate records were removed with the support of EndNote reference management software. Following this initial screening, the full texts of potentially eligible studies were reviewed to confirm compliance with the predefined inclusion and exclusion criteria.

Data extraction was conducted independently by the same reviewers using a standardized data collection form. Detailed information was collected on the methodological and clinical characteristics of the included studies, namely: study identification (authors, year), participants’ mean age (and standard deviation), gender distribution (male/female), total and group-specific sample size, and the average stage of PD according to the Hoehn and Yahr scale. Regarding the interventions, the experimental group (EG) protocol, the control group (CG) comparator, and the dosage (frequency, session duration, and total intervention period) were described. The assessed outcomes were recorded and categorized as primary or secondary, along with the instruments used for their measurement and the main findings. For each outcome (continuous variables), post-intervention means, and standard deviations were extracted for each group, as well as the number of participants per group, in order to enable the calculation of between-group mean differences and corresponding 95% confidence intervals (CIs), for descriptive purposes. Any reported adverse events were also recorded.

Any discrepancies between reviewers were settled by consensus or, when necessary, with input from a third reviewer. Studies with incomplete or inaccessible relevant data were excluded from the final analysis.

### 2.5. Eligibility Criteria

Studies were eligible for inclusion if they met all of the following criteria: (1) RCTs; (2) published between 1 January 2019, and 23 April 2025; (3) full text available in Portuguese or English; (4) participants diagnosed with PD at stages 1 to 4 on the Hoehn and Yahr scale; (5) EG receiving interventions involving VR, either alone or combined with conventional physiotherapy; (6) CG receiving conventional physiotherapy only; and (7) assessment of outcomes related to balance and/or gait, using at least one validated measurement tool for either domain.

Studies were excluded if they met any of the following criteria: (1) study design other than RCTs (e.g., systematic reviews, cohort studies, case–control studies, case reports, case series, editorials, or expert opinions); (2) grey literature; (3) published before 2019; (4) animal studies; (5) participants without a confirmed diagnosis of PD; (6) interventions exclusively pharmacological or surgical; or (7) retracted or withdrawn publications.

### 2.6. Methodological Quality Assessment and Risk of Bias

The methodological quality of the included studies was assessed using the Portuguese version of the PEDro scale [20], a validated and widely adopted tool for evaluating clinical trials in the field of physiotherapy. This scale consists of 11 items, of which 10 contribute to the final score, since item 1, related to external validity, is reported but not scored. The criteria assessed include specification of eligibility criteria, random allocation, concealed allocation, similarity of groups at baseline, blinding of participants, blinding of therapists, blinding of assessors, availability of outcome data for at least 85% of participants, intention-to-treat analysis, between-group statistical comparisons, and reporting of point estimates with measures of variability. The total score ranges from 0 to 10 points, with scores of 9–10 indicating excellent methodological quality, 6–8 good, 4–5 fair, and 3 or less poor quality. The assessment was conducted independently by four reviewers working in pairs. Any disagreements were resolved by consensus or through consultation with a third reviewer. This process enabled the identification of potential sources of bias and supported a more rigorous and robust interpretation of the findings.

### 2.7. Data Synthesis and Analysis

The collected data were organized into comparative tables presenting means, standard deviations, mean differences between EG and CG, as well as CIs for primary and secondary outcomes. Only one data conversion was performed during the review: one study reported values as medians and interquartile ranges, which were converted to means and standard deviations using the online calculator available at https://www.math.hkbu.edu.hk/~tongt/papers/median2mean.html, (accessed on 7 April 2025) to ensure consistency with the remaining studies. For calculating the mean difference and the respective CI between groups, the online calculator provided at https://www.statskingdom.com/difference-confidence-interval-calculator.html (accessed on 7 April 2025) was used.

Given the limited number of included studies and the heterogeneity of intervention protocols and outcome measures, a formal meta-analysis was not conducted to avoid potential biases and inappropriate statistical interpretations. The synthesis performed, of a descriptive and simple quantitative nature, allows the assessment of trends and variations in the effects of VR interventions, either alone or combined with conventional physiotherapy. Formal analyses of heterogeneity, sensitivity, or publication bias were not carried out due to the small number of studies and methodological diversity. However, the rigorous assessment of methodological quality was considered in interpreting the results.

For analytical clarity, the included studies were grouped into two categories according to the type of experimental intervention: (1) studies in which VR was applied as a stand-alone intervention, and (2) studies in which VR was combined with conventional physiotherapy. This allowed for a clearer comparison of the differential effects of each intervention modality.

### 2.8. Certainty of Evidence Assessment

The certainty of the evidence was qualitatively assessed considering five criteria: risk of bias, consistency of results, precision of estimates, magnitude of observed effects, and clinical applicability. To ensure transparency and minimize bias, four reviewers, in pairs, carried out this assessment, addressing any disagreements by consensus or by involving a third reviewer. No evidence of publication bias was identified based on the available information.

## 3. Results

### 3.1. Study Selection Process

**Figure 1:**
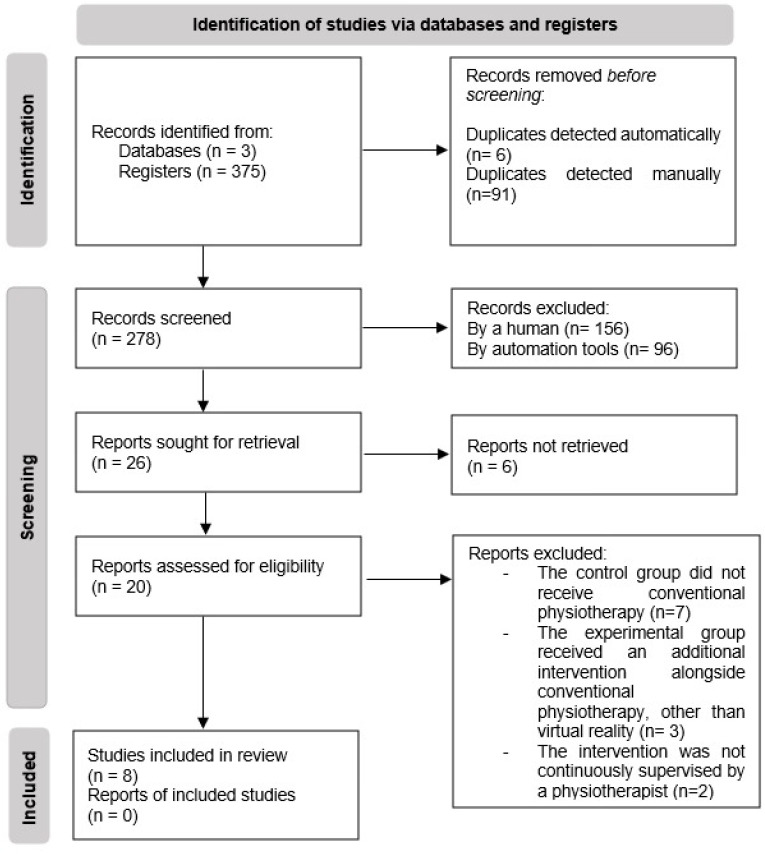
PRISMA Flow Diagram of Study Selection Process.

The PRISMA flow diagram (Figure 1) outlines the systematic process of study selection, encompassing the phases of identification, screening, and inclusion. A total of 375 records were initially identified across three databases: 111 from PubMed, 231 from Web of Science, and 33 from PEDro. After the removal of 97 duplicates (6 automatically and 91 manually), 278 records remained for title and abstract screening. Of these, 252 were excluded: 96 by automated filtering in EndNote based on publication date, and 156 by manual screening for relevance conducted by reviewers. The remaining 26 reports were sought for full-text retrieval, but 6 could not be obtained. Thus, 20 reports were assessed for eligibility. Of these, 12 were excluded for the following reasons: in 7 studies, the CG did not receive conventional physiotherapy; in 3, the EG received an additional intervention other than VR; and in 2, the intervention was not continuously supervised by a physiotherapist. As a result, 8 studies met the inclusion criteria and were included in the systematic review. No additional eligible reports were identified for these studies. Figure 1 summarizes the study selection process, ensuring transparency and methodological rigor in accordance with PRISMA guidelines.

### 3.2. Methodological Quality of Included Studies

The methodological quality of the included RCTs was assessed using the PEDro scale, with scores ranging from 5 to 7 points out of a possible maximum of 10 (Table 1). All studies met the criteria for random allocation of participants (item #2), baseline group similarity (item #4), blinding of assessors (item #7), statistical comparisons between groups (item #10), and presentation of point estimates accompanied by measures of variability (item #11). Item #1 (participant eligibility criteria) was also met by almost all studies.

None of the studies ensured the blinding of participants (item #5) or therapists (item #6). Allocation concealment (item #3), collection of data from more than 85% of initially randomized participants (item #8), and the application of intention-to-treat analysis (item #9) were inconsistently implemented.

Studies included in Group I (intervention with isolated VR training) presented scores between 5 and 7, with the works by Feng et al. [12], Da Silva et al. [16], and Nuvolini et al. [19] achieving the highest score (7/10). In Group II (VR combined with conventional physiotherapy), scores ranged from 6 to 7, indicating a globally comparable methodological profile to Group I.

### 3.3. Characteristics of Studies Using VR-Only Interventions

Table 2 presents the main characteristics of the six studies that evaluated interventions based exclusively on VR in individuals with PD. Sample sizes ranged from 20 to 51 participants, with a balanced gender distribution. The mean age of participants varied between 62.7 and 72.0 years. Most studies included patients at stages 1 to 3 of the Hoehn and Yahr scale, although some variation was observed in how these data were reported. The experimental interventions consisted of VR training protocols, either immersive or non-immersive, with session durations ranging from 20 to 60 min, applied 2 to 5 times per week over periods of 5 to 12 weeks. CG generally receives conventional physiotherapy, including physical activity programs. The assessed measures focused primarily on balance and gait, such as the BBS, TUGT, 10MWT, and UPDRS III. Some studies also included secondary assessments of function (DASH, 5xSTS and FIM), cognition (MoCA) and quality of life (PDQ-39 and SF-36). Adverse event monitoring was conducted in two studies, with no significant side effects observed.

### 3.4. Characteristics of Studies Combining VR and Conventional Physiotherapy

Table 3 summarizes the main characteristics of the two included studies that evaluated interventions combining VR training with conventional physiotherapy in people with PD. Sample sizes ranged from 30 to 60 participants, with a relatively balanced gender distribution. The mean age of participants ranged from 61.95 to 75.5 years, with most patients in stages 2 to 3 of the Hoehn and Yahr scale. Experimental interventions involved combined protocols of VR training and traditional physiotherapy, applied 2 to 3 times per week, with sessions lasting 50 to 60 min, over periods ranging from 5 to 12 weeks. CG received conventional physiotherapy only. Outcome measures primarily included key balance and gait parameters, such as BBS, ABCS, FES-I, Gait Speed, POMA and UPDRS III. Secondary outcomes covered function and quality of life, including BI, UPDRS II and SF-12. Monitoring of adverse effects, such as nausea, dizziness, and vertigo, was reported in one study, with no significant side effects observed.

### 3.5. Types of Virtual Reality and Devices Used

The eight RCTs included in this review employed a range of virtual reality modalities and hardware platforms. Five studies implemented non-immersive VR: Kashif et al. [15] used the Nintendo Wii console, controllers, and Fit board; Feng et al. [12] applied a monitor-based “pull the body” screen; Da Silva et al. [16] utilized Xbox Kinect exergames; Maranesi et al. [17] used a screen-based platform for virtual exergames; and Nuvolini et al. [19] relied on randomized play of four Xbox 360 Kinect Adventures games. In contrast, immersive VR was employed by Schuch et al. [18] via the mobile VR Box^®^ smartphone headset, and by Pazzaglia et al. [13] through the NIRVANA markerless optoelectronic system. Pullia et al. [14] adopted a semi-immersive VR approach, integrating a C-Mill treadmill with VR projection.

### 3.6. Primary Outcomes: VR-Only Interventions

Table 4 presents the post-intervention means of the primary outcomes, sample sizes, mean differences between EG and CG, and the corresponding 95% CIs, in studies that applied VR training exclusively.

In the study by Feng et al. [12], the EG had a mean of 36.71 points on the BBS compared to 32.00 in the CG, with a mean difference of +4.71 points (95% CI [1.05; 8.37]). For the FGA, the EG scored 21.21 points versus 18.43 in the CG (+2.78; 95% CI [0.02; 5.54]). In the TUGT, the mean was 30.93 s in the EG and 35.14 s in the CG, corresponding to a difference of −4.22 s (95% CI [−8.34; −0.08]). In these three outcomes, the CI did not include null value, suggesting statistically significant between-group differences. Conversely, in the UPDRS III, the between-group difference was +0.14 points (95% CI [−5.69; 5.97]); the confidence interval includes the null value, suggesting that this difference is not statistically significant.

In Pazzaglia et al. [13], the mean difference on the BBS was +1.1 points (95% CI [−3.21; 5.41]) and on the DGI +1.2 points (95% CI [−1.08; 3.48]); in both cases, the CIs include the null value, suggesting a lack of statistical significance.

Schuch et al. [18] reported a mean of 1.10 m/s for the 10MWT in the EG versus 1.00 m/s in the CG, with a difference of +0.10 m/s (95% CI [0.05; 0.15]). For the TUGT, the meantime post-intervention was 9.80 s in the experimental group and 10.80 s in the control group, resulting in a difference of −1.0 s (95% CI [−1.48; −0.52]). Here, the CIs exclude null value, suggesting statistically significant differences.

Da Silva et al. [16] reported non-significant differences between groups for the Mini-BESTest (+0.56 points; 95% CI [−2.22; 3.34]), LOS (+8.81 cm^2^; 95% CI [−27.17; 44.79]), ABCS (+5.29%; 95% CI [−6.49; 17.07]), TUGT (−0.25 s; 95% CI [−3.06; 2.56]), and UPDRS III (−1.13 points; 95% CI [−5.89; 3.63]), as the CIs include the null value.

Pullia et al. [14] reported a mean score of 52.44 points on the BBS in the EG versus 32.55 in the CG, corresponding to a difference of +19.89 points (95% CI [0.25; 39.53]); a mean of 16.11 points on the FES-I in the EG versus 32.44 in the CG (−16.33 points; 95% CI [−31.93; −0.73]); and a 6MWT distance of 460.88 m in the EG compared to 247.75 m in the CG (+213.13 m; 95% CI [43.90; 382.36]). All these confidence intervals exclude null value, suggesting statistically significant differences. However, for other outcomes such as the 10MWT, TUGT for right and left side tests, POMA, and UPDRS III, the CIs include null value, indicating no significant differences.

Finally, Nuvolini et al. [19] reported non-significant differences between groups in several outcomes. For the 10MWT in single-task conditions, the difference was 0 (95% CI [−0.24; 0.24]). In dual-task conditions, the difference was +0.08 m/s (95% CI [−0.07; 0.23]). For the FGA, the difference was +1.30 points (95% CI [−1.63; 4.23]). In the TUGT, the difference was −0.5 s (95% CI [−3.37; 2.37]). In all these cases, the confidence intervals include the null value, suggesting that the differences were not statistically significant.

### 3.7. Primary Outcomes: Combined VR and Physiotherapy Interventions

Table 5 presents post-intervention means for primary outcomes, sample sizes, mean differences between the EG and CG, and the corresponding 95% CIs, from studies that combined VR with conventional physical therapy.

In the study by Maranesi et al. [17], the mean difference in the FES-I score was −0.8 points (95% CI [−2.00, 0.40]). Gait speed showed a mean difference of +0.1 m/s (95% CI [−0.36, 0.56]). In both outcomes, the CIs include the null value, suggesting that the between-group differences are not statistically significant. For the POMA, the EG achieved 25.9 points versus 23.3 in the CG, with a mean difference of +2.6 points (95% CI [1.70, 3.50]); in this case, the CI does not include the null value, indicating statistical significance.

In the study by Kashif et al. [15], the mean BBS score was 50.1 in the EG and 45.5 in the CG, with a mean difference of +4.6 points (95% CI [1.57, 7.63]). For the ABCS, the EG had a mean of 78.59% compared to 71.56% in the CG, yielding a difference of +7.03% (95% CI [2.36, 11.70]). In the UPDRS III, the EG scored 17.20 points versus 24.45 in the CG, with a mean difference of −7.25 points (95% CI [−12.15, −2.35]). In all these outcomes, the CIs exclude the null value, suggesting statistically significant differences between groups.

### 3.8. Secondary Outcomes: VR-Only Interventions

Table 6 presents the post-intervention means of the secondary outcomes, sample sizes, mean differences between the EG and the CG, and the corresponding 95% CIs, for the studies that used VR interventions exclusively. Only four of the six studies included in this group reported secondary outcomes with numerical data.

In the study by Pazzaglia et al. [13], the difference in the DASH was −3.5 points (95% CI [−12.2; 5.2]). In the physical domain of the SF-36, the EG scored 36.8 points versus 46.7 in the CG, with a difference of −9.9 points (95% CI [−16.57; −3.23]). In the mental domain of the SF-36, the difference was +4.3 points (95% CI [−1.93; 10.53]). Only the physical domain of the SF-36 showed a CI that excluded the null value, suggesting a statistically significant between-group difference.

In the study by Da Silva et al. [16], the difference in the 5xSTS was −1.62 s (95% CI [−5.03; 1.79]). On the PDQ-39, the difference was +2.77 points (95% CI [−7.05; 12.59]). In both outcomes, the CIs included the null value, indicating a lack of statistical significance.

In the study by Pullia et al. [14], the mean FIM score was 120.57 in the EG and 99.33 in the CG, with a difference of +21.24 points (95% CI [3.18; 39.3]), suggesting a statistically significant difference in favor of the EG.

Finally, in the study by Nuvolini et al. [19], the difference in MoCA scores was −0.6 points (95% CI [−3.36; 2.16]). The CI included the null value, indicating no statistically significant difference.

### 3.9. Secondary Outcomes: Combined Interventions

Table 7 presents the post-intervention means of the secondary outcomes, sample sizes, mean differences between the EG and the CG, and the corresponding 95% CIs, in the two studies that combined VR with conventional physiotherapy.

In the study by Maranesi et al. [17], the EG had a mean score of 94.3 points on the Barthel Index, compared to 87.6 in the CG, resulting in a mean difference of +6.7 points (95% CI [3.63; 9.77]). This CI excludes the null value, indicating a statistically significant difference in favor of the EG. Conversely, for the SF-12, the difference was −0.2 points (95% CI [−0.69; 0.29]), a CI that includes the null value and does not indicate statistical significance.

In the study by Kashif et al. [15], the UPDRS II revealed a statistically significant difference of −4.2 points in favor of the EG (95% CI [−6.19; −2.21]), with post-intervention means of 15.30 and 19.50 points in the EG and CG, respectively.

### 3.10. Certainty of the Evidence

The certainty of the evidence assessment considered five criteria.

Regarding risk of bias, most studies presented in Table 1 demonstrated fair to good methodological quality, indicating a moderate risk of bias.

The consistency of results varied across studies and outcomes, as shown in Table 4, Table 5, Table 6 and Table 7. Among the 31 comparisons related to primary outcomes (Table 4 and Table 5), 30 showed a mean difference favoring the experimental group, with 12 reaching statistical significance; only one comparison favored the control group, without statistical significance. For secondary outcomes (Table 6 and Table 7), 6 out of 10 comparisons favored the experimental group (3 statistically significant), while 4 favored the control group (1 statistically significant).

Regarding precision, 16 CIs excluded the null value, indicating precise and statistically significant estimates; conversely, 25 CIs were wide and included the null value, reflecting lower precision and absence of statistical significance.

The magnitude of observed effects ranged from small to moderate across most outcomes. However, some comparisons demonstrated large effects, such as the BBS (mean difference up to +19.9 points) and the 6MWT (mean difference of +213.1 m) in the study by Pullia et al. [14]. In contrast, several outcomes showed small or non-significant effects, such as those reported by Da Silva et al. [16] and Nuvolini et al. [19], where no statistically significant differences were observed.

Finally, the clinical applicability of the findings may be limited by heterogeneity in intervention protocols and sample characteristics, as detailed in Table 2 and Table 3 and described earlier.

## 4. Discussion

This systematic review aimed to evaluate the effects of VR-based interventions, applied either alone or combined with conventional physiotherapy, on primary outcomes of balance and gait in people with PD. In addition to this, secondary outcomes related to function, cognition, and quality of life were also considered.

The study selection process, conducted according to PRISMA guidelines and based on clearly defined eligibility criteria, resulted in the inclusion of only eight RCTs, six using VR alone and two integrating VR with conventional physiotherapy, from an initial pool of 375 records identified. The strict application of inclusion criteria contributed to this limited number, reflecting not only a scarcity of research in comparable clinical settings but also notable methodological heterogeneity across the available studies. Accordingly, these findings should be interpreted with caution, given the relatively small and heterogeneous body of evidence, which constrains their generalizability.

Methodological quality was rated as fair to good, with PEDro scores ranging from 5 to 7. Overall, key aspects such as random allocation of participants, baseline group equivalence, assessor blinding, statistical comparisons between groups, presentation of point estimates accompanied by measures of variability, and clear sample definitions based on eligibility criteria were generally consistent. However, none of the studies ensured blinding of participants or therapists, a limitation inherent to the design of physiotherapy interventions. Significant methodological weaknesses also persisted regarding allocation concealment, collection of data from at least 85% of initially randomized participants, and the use of intention-to-treat analysis. These factors increase the risk of bias and must be carefully considered when critically interpreting the results.

Regarding the primary outcomes, balance measures such as the BBS, ABCS, and FES-I, gait measures including the 10MWT, 6MWT, FGA, and TUGT, as well as more global assessments like the POMA and UPDRS III, were frequently evaluated. Some studies applying interventions based solely on VR demonstrated statistically significant improvements. In the study by Pullia et al. [14], gains of 19.89 points on the BBS, 16.33 points on the FES-I, and 213.13 m on the 6MWT were reported. These values exceed the minimal detectable change (MDC) thresholds for each measure (5 points [21], 9.6 points [22], and 82 m [21], respectively), indicating possible clinical relevance, albeit limited to the context of this particular study. This study stands out from the others by employing a semi-immersive VR system integrated into a treadmill-based gait training protocol (C-Mill), which may have contributed to the observed superior effects compared to conventional physiotherapy. However, it is worth noting that this trial was among those with the lowest PEDro scores in the review, having received 5 out of 10 points, which should be considered when interpreting its results. Conversely, Feng et al. [12] observed statistically significant improvements favoring isolated VR interventions, including 4.71 points on the BBS, 2.78 points on the FGA, and 4.22 s on the TUGT, which nonetheless fell below the respective MDC thresholds of 5 points [21], 4 points [23], and 11 s [21]. Similarly, Schuch et al. [18] reported significant gains with isolated VR, but these did not reach clinical relevance, showing a reduction of only 1 s on the TUGT (MDC = 11 s [21]) and an increase of 0.1 m/s in comfortable walking speed on the 10MWT (MDC = 0.18 m/s [21]). Conversely, interventions combining VR with conventional physiotherapy showed statistically significant improvements that approached or slightly missed the clinical thresholds. Maranesi et al. [17] documented a 2.6-point improvement on the POMA (MDC = 2.8 points [24]), while Kashif et al. [15] reported gains of +4.6 points on the BBS (MDC = 5 points [21]), +7.03% on the ABCS (MDC = 13% [25]), and a reduction of −7.25 points on the UPDRS III (MDC = 11 points [21]). These findings suggest positive trends, although their clinical impact should be interpreted cautiously, particularly given the heterogeneity of the studies, limited consistency of results, and the small number of eligible investigations included in this review. Moreover, it is important to highlight that only 38.7% of the comparisons related to the primary outcomes showed statistical significance, underscoring the need for cautious interpretation of the observed effects.

Secondary outcomes were less frequent and heterogeneous, which complicated direct comparisons between studies. Nevertheless, some functional measures showed significant improvements favoring VR intervention. In the study by Pullia et al. [14], the FIM score increased by 21.24 points. In the study by Maranesi et al. [17], the Barthel Index improved by 6.7 points in favor of the intervention combining VR with physiotherapy, and in the study by Kashif et al. [15], gains of 4.2 points were observed in the UPDRS II (MDC = 4 [21]), also favoring the experimental group. Regarding quality of life, only the physical domain of the SF-36 in the study by Pazzaglia et al. [13] showed statistical significance, with the group receiving VR-only intervention scoring 9.9 points lower. No other statistically significant differences were observed, particularly in cognition. Despite the need for caution when interpreting these results, this pattern suggests that the benefits of VR tend to be more consistent in functional domains than in cognitive or quality of life-related areas. Nonetheless, the limited follow-up durations reported preclude any conclusions about long-term benefits, an issue that must be addressed in future trials.

The qualitative assessment of the certainty of evidence was based on five key criteria: risk of bias, consistency of results, precision of estimates, magnitude of effects, and clinical applicability. The risk of bias was deemed moderate, reflecting the fair to good methodological quality of most included studies. Consistency varied depending on the outcome, with most primary outcomes favoring the experimental group, whereas secondary outcomes showed greater heterogeneity. Regarding precision, fewer than half of the estimates excluded the null value, pointing to wide confidence intervals and, consequently, statistical uncertainty. The magnitude of effects ranged from small to moderate, with a few notable exceptions. Clinical applicability was limited due to variability in intervention protocols and sample characteristics, which restricts the generalizability of findings. Moreover, the wide heterogeneity in VR systems—ranging from immersive environments to non-immersive platforms—adds further complexity, as it remains unclear which technological features are most beneficial or cost-effective. These factors underscore the urgent need for future studies with more rigorous and robust methodological designs, including larger sample sizes to enhance statistical power. Standardizing outcomes and their respective measures is equally critical, as it will enable more consistent comparisons across studies and support the development of meaningful meta-analyses. In addition, harmonizing intervention protocols—particularly regarding the type of VR (immersive vs. non-immersive), total intervention time, session duration, frequency, and supervision—would help reduce heterogeneity and foster more reliable comparisons, even if the most effective combination of parameters has yet to be determined. A systematic and transparent approach to reporting data should become a priority. This includes ensuring proper allocation concealment, retaining a representative proportion of participants throughout the study, and conducting analyses that follow the intention-to-treat principle. These methodological practices are essential to minimize bias and enhance the internal validity of findings. Finally, incorporating standardized instruments that assess functional, cognitive, and quality-of-life domains simultaneously will allow for a more comprehensive understanding of the clinical impact of VR in PD.

In summary, VR-based interventions show promise for improving balance and gait in people with PD, particularly when integrated into conventional physiotherapy programs. However, the magnitude and consistency of the observed effects do not yet allow us to confidently assert the superiority of this approach over traditional methods. The interpretation of between-group differences in primary outcomes was based on clinically established thresholds for MDC. Therefore, results from these instruments were analyzed descriptively and with increased caution. In this review, MDC values were used solely as interpretative references to aid in understanding the extent of the observed effects. This approach aims to ensure consistent and objective interpretation, avoiding selective emphasis on outcomes exceeding MDC thresholds. It is also important to highlight the motivating and interactive potential of VR, which may enhance patient adherence and engagement in rehabilitation programs. Nonetheless, the benefits of VR when used in isolation appear limited, with its greatest effectiveness likely stemming from combination with conventional interventions in programs supervised by physiotherapists. Despite its motivational appeal, the current evidence does not justify its widespread use as a standalone intervention. This perspective is supported by the literature. The review by Lei et al. [10] shows that VR-based rehabilitation can achieve results comparable to conventional training, particularly in parameters such as gait and balance, and may even outperform traditional methods in some cases. Thus, when traditional training proves insufficient, VR emerges as a valid alternative. Still, as evidenced in this analysis, there remains an urgent need for more rigorous studies with larger samples and multicenter designs to deepen understanding of VR’s potential benefits. Important gaps persist, including the lack of consensus on the ideal type of training, heterogeneity of protocols used, and scarcity of programs tailored to the different stages of the disease. The ongoing advancement of sensor-based technologies and VR platforms opens new avenues in neurorehabilitation. Solutions enabling precise remote monitoring and personalized home-based interventions supported by real-time data could play a central role in the rehabilitation of people with PD. However, the absence of long-term data, underrepresentation of disease stages, and methodological inconsistencies across trials must be resolved before VR can be confidently integrated into routine clinical practice. Overall, the patterns observed in this review indicate a relatively consistent trend favoring VR interventions. Of the 31 comparisons related to primary outcomes, 30 favored the experimental group, with 12 reaching statistical significance. Only one comparison favored the control group, without statistical significance. Regarding secondary outcomes, 6 out of 10 comparisons favored the experimental group (3 statistically significant), while 4 favored the control group (1 significant). Although these trends appear promising, their clinical and statistical robustness remains fragile, and definitive claims should be withheld until higher-quality evidence becomes available.

## 5. Conclusions

This systematic review suggests that VR-based interventions may have a positive effect on balance and gait in people with PD, particularly when integrated into conventional physiotherapy. However, the limited number of heterogeneous studies, combined with methodological limitations and inconsistency in the clinical relevance of outcomes, calls for cautious interpretation. The evidence indicates that the benefits of VR seem to be more closely linked to the dosage and integration of these interventions rather than their isolated use. The wide variability in VR types, protocols, and outcome measures complicates the drawing of definitive conclusions, underscoring the urgent need for more rigorous RCTs with greater statistical power and standardization. Future research should focus on harmonizing intervention parameters, adopting robust methodologies, and extending follow-up periods to clarify long-term effects. Moreover, the motivational and interactive aspects of VR may enhance patient adherence, although current evidence does not support its widespread use as a standalone treatment. VR should be regarded as a promising adjunct to comprehensive physiotherapy programs tailored to the stage of PD and individual patient needs. Ultimately, the consolidation of VR as an effective, safe, and accessible tool in Parkinson’s rehabilitation will depend on the generation of consistent, high-quality evidence supporting its clinical integration in real-world settings.

## Figures and Tables

**Table 1:** Methodological Quality Ratings of the Included Randomized Controlled Trials According to the PEDro Scale.

Individual Item Ratings and Total PEDro Score												
Study	#1 Eligibility Criteria Specified	#2 Random Allocation	#3 Concealed Allocation	#4 Groups Similar at Baseline	#5 Blinding of Subjects	#6 Blinding of Therapists	#7 Blinding of Assessors	#8 Outcome Data from >85% of Subjects	#9 Intention-to-Treat Analysis	#10 Reported Between-Group Comparisons	#11 Point Estimates and Variability Measures	Total
**Group I: Virtual Reality Training Only**												
Feng et al., 2019 [12]	1	1	0	1	0	0	1	1	1	1	1	7/10
Pazzaglia et al., 2020 [13]	0	1	0	1	0	0	1	0	0	1	1	5/10
Schuch et al., 2020 [18]	1	1	0	1	0	0	1	1	0	1	1	6/10
Da Silva et al., 2023 [16]	1	1	1	1	0	0	1	0	1	1	1	7/10
Pullia et al., 2023 [14]	1	1	0	1	0	0	1	0	0	1	1	5/10
Nuvolini et al., 2025 [19]	1	1	1	1	0	0	1	0	1	1	1	7/10
**Group II: Virtual Reality Training Combined with Physical Therapy**												
Maranesi et al., 2022 [17]	1	1	0	1	0	0	1	1	1	1	1	7/10
Kashif et al., 2024 [15]	1	1	0	1	0	0	1	1	0	1	1	6/10

Note: Item 1 (Eligibility criteria specified) is not included in the total PEDro score, as it relates to external validity rather than the methodological quality of the trial.

**Table 2:** Main Characteristics of the Included Studies on Isolated Virtual Reality Interventions.

Group I: Virtual Reality Training Only								
Study	Age (Mean ± SD) Gender (M/F)	Sample Size	EG	CG	Hoehn-Yahr (Mean ± SD or Frequency)	Dosage	Outcomes	Adverse Events
Feng et al., 2019 [12]	EG: 67.47 ± 4.79 (8/7) CG: 66.93 ± 4.64 (9/6)	28	VR training	Conventional physical therapy	EG: 3.03 ± 0.55 CG: 2.97 ± 0.58	45 min/day 5 days/week 12 weeks	BBS, FGA, TUGT, UPDRS III	m.d
Pazzaglia et al., 2020 [13]	EG: 72.0 ± 7.0 (18/7) CG: 70.0 ± 10.0 (17/9)	51	VR training	Conventional physical therapy	m.d	40 min/day 3 days/week 6 weeks	BBS, DGI, DASH, SF-36	m.d
Schuch et al., 2020 [18]	EG: 63.0 ± 2.8 (6/5) CG: 69.1 ± 2.3 (10/2)	23	VR training	Physical activities (walking, stretching exercises) + Psycho Education	EG: Stage 1-1 Stage 1.5-2 Stage 2-4 Stage 2.5-4 Stage 3-0 CG: Stage 1-0 Stage 1.5-4 Stage 2-3 Stage 2.5-1 Stage 3-4	20 min/day 2 days/week 5 weeks	10MWT, TUGT	Cybersickness, dizziness, nausea, and falls were monitored during each session.
Da Silva et al., 2023 [16]	EG: 63.3 ± 6.46 (14/4) CG: 68.0 ± 10.2 (19/1)	38	Kinect exergame-based training	Conventional physiotherapy training	EG: Stage 1-3 Stage 1.5-4 Stage 2-3 Stage 2.5-3 Stage 3-5 CG: Stage 1-2 Stage 1.5-7 Stage 2-4 Stage 2.5-4 Stage 3-3	60 min/day 2 days/week 7 weeks	Mini-BESTest, LOS, ABCS, TUGT, UPDRS III, 5xSTS, PDQ-39	m.d
Pullia et al., 2023 [14]	EG: 64.5 ± 10.84 (9/1) CG: 65.5 ± 10.36 (4/6)	20	The C-Mill Gait Training	Conventional rehabilitation treatments	EG: 2.5 ± 0.78 CG: 3.5 ± 0.36	45 min/day 4 days/week 5 weeks	BBS, FES-I, 10MWT, 6MWT, TUGT, POMA, UPDRS III, FIM	All patients completed the training without reporting any adverse effects, including cybersickness.
Nuvolini et al., 2025 [19]	EG: 62.7 ± 6.8 (15/4) CG: 69.2 ± 7.8 (18/1)	38	Kinect exergame-based training	Conventional physiotherapy	EG: Stage 1-3 Stage 1.5-4 Stage 2-4 Stage 2.5-3 Stage 3-5 CG: Stage 1-1 Stage 1.5-6 Stage 2-4 Stage 2.5-5 Stage 3-3	60 min/day 2 days/week 7 weeks	10MWT, FGA, TUGT, MoCA	m.d

Legend: 10MWT—10-Meter Walk Test; 5xSTS—Five Times Sit-to-Stand Test; 6MWT—6-Minute Walking Test; ABCS—Activities-specific Balance Confidence Scale; BBS—Berg Balance Scale; CG—Control Group; DASH—Disabilities of the Arm, Shoulder and Hand Questionnaire; DGI—Dynamic Gait Index; EG—Experimental Group; F—Female; FES-I—Falls Efficacy Scale-International; FGA—Functional Gait Assessment; FIM—Functional Independence Measure; LOS—Limit of Stability; M—Male; Mini-BESTest—Mini Balance Evaluation Systems Test; m.d—Missing Data; MoCA—Montreal Cognitive Assessment; PDQ-39—Parkinson’s Disease Questionnaire; POMA—Performance Oriented Mobility Assessment; SD—Standard Deviation; SF-36—36-Item Short Form Health Survey; TUGT—Timed Up and Go Test; UPDRS III—The third part of Unified Parkinson’s Disease Rating Scale; VR—Virtual Reality.

**Table 3:** Main Characteristics of the Included Studies on Virtual Reality Combined with Physiotherapy.

Group II: Virtual Reality Training Combined with Physical Therapy								
Study	Age (Mean ± SD) Gender (M/F)	Sample Size	EG	CG	Hoehn-Yahr (Mean ± SD)	Dosage	Outcomes	Adverse Events
Maranesi et al., 2022 [17]	EG: 72.7 ± 6.3 (6/10) CG: 75.5 ± 5.4 (9/5)	30	Tymo system + Traditional therapy	Traditional therapy sessions	EG: 2.0 ± 0.8 CG: 2.3 ± 0.9	50 min/day 2 days/week 5 weeks	FES-I, Gait Speed *, POMA, BI, SF-12	m.d
Kashif et al., 2024 [15]	EG I: 63.20 ± 4.85 (12/8) EG II: 64.85 5.10 (10/10) CG: 61.95 + 4.62 (11/9	60	EG I: VR training + Physical therapy EG II: Motor Imagery + Physical therapy	Physical therapy	EG I: 2.07 ± 0.75 EG II: 2.10 ± 0.61 CG: 2.32 ± 0.63	60 min/day 3 days/week 12 weeks	BBS, ABCS, UPDRS III, UPDRS II	Common side effects of virtual reality, such as nausea, dizziness, and vertigo, often referred to as cybersickness or simulator sickness, were monitored.

Legend: ABCS—Activities-specific Balance Confidence Scale; BI—Barthel Index; BBS—Berg Balance Scale; CG—Control Group; EG—Experimental Group; F—Female; FES-I—Falls Efficacy Scale-International; M—Male; m.d—Missing Data; POMA—Performance Oriented Mobility Assessment; SD—Standard Deviation; SF-12—12-Item Short Form Health Survey; UPDRS II—The second part of Unified Parkinson’s Disease Rating Scale; UPDRS III—The third part of Unified Parkinson’s Disease Rating Scale; VR—Virtual Reality. * Test distance or protocol not specified.

**Table 4:** Primary Outcomes—Post-Intervention Means, Sample Sizes, Mean Differences, and Confidence Intervals from Studies Using Virtual Reality Interventions.

Group I: Virtual Reality Training Only					
Study	Outcome	Group	Mean (SD) Post-Intervention	*n*	Mean Difference (95% CI) *
Feng et al., 2019 [12]	BBS (points)	EG	36.71 (4.60)	14	**+4.71** **[1.05; 8.37]**
		CG	32.00 (4.82)	14	
	FGA (points)	EG	21.21 (3.95)	14	**+2.78** **[0.02; 5.54]**
		CG	18.43 (3.09)	14	
	TUGT (s)	EG	30.93 (5.55)	14	**−4.22** **[−8.34; −0.08]**
		CG	35.14 (5.07)	14	
	UPDRS III (points)	EG	21.50 (6.81)	14	+0.14 ^‡^ [−5.69, 5.97]
		CG	21.36 (8.15)	14	
Pazzaglia et al., 2020 [13]	BBS (points)	EG	49.2 (8.1)	25	+1.1 [−3.21, 5.41]
		CG	48.1 (7.2)	26	
	DGI (points)	EG	20.2 (4.2)	25	+1.2 [−1.08, 3.48]
		CG	19.0 (3.9)	26	
Schuch et al., 2020 [18]	10MWT (m/s)	EG	1.10 (0.07)	11	**+0.1** **[0.05; 0.15]**
		CG	1.00 (0.05)	12	
	TUGT (s)	EG	9.80 (0.60)	11	**−1.0** **[−1.48; −0.52]**
		CG	10.80 (0.50)	12	
Da Silva et al., 2023 [16]	Mini-BESTest (points)	EG	22.56 (4.09)	18	+0.56 [−2.22; 3.34]
		CG	22.00 (4.34)	20	
	LOS (cm^2^)	EG	146.41 (59.96)	18	+8.81 [−27.17, 44.79]
		CG	137.60 (49.31)	20	
	ABCS (%)	EG	70.97 (15.78)	18	+5.29 [−6.49, 17.07]
		CG	65.68 (19.57)	20	
	TUGT (s)	EG	11.32 (4.84)	18	−0.25 [−3.06; 2.56]
		CG	11.57 (3.68)	20	
	UPDRS III (points)	EG	16.17 (7.70)	18	−1.13 [−5.89, 3.63]
		CG	17.30 (6.76)	20	
Pullia et al., 2023 [14]	BBS (points)	EG	52.68 (4.30) **	10	**+19.89** **[0.25; 39.53]**
		CG	32.79 (29.24) **	10	
	FES-I (points)	EG	26.14 (9.89) **	10	**−16.33** **[−31.93, −0.73]**
		CG	42.47 (21.29) **	10	
	10MWT (m/s)	EG	1.98 (0.61) **	10	+0.27 [−0.85; 1.39]
		CG	1.71 (1.57) **	10	
	6MWT (m)	EG	362.22 (30.96) **	10	**+213.13** **[43.90; 382.36]**
		CG	149.09 (252.84) **	10	
	TUGT right (s)	EG	9.44 (4.39) **	10	−4.56 [−15.77; 6.65]
		CG	14.00 (16.29) **	10	
	TUGT left (s)	EG	9.54 (3.63) **	10	−4.02 [−14.20; 6.16]
		CG	13.56 (14.88) **	10	
	POMA (points)	EG	26.08 (3.87) **	10	+8.17 [−1.60; 17.94]
		CG	17.91 (14.19) **	10	
	UPDRS III (points)	EG	28.46 (25.37) **	10	−3. 04 [−23.17, 17.09]
		CG	31.50 (16.56) **	10	
Nuvolini et al., 2025 [19]	10MWT/ST (m/s)	EG	1.33 (0.37)	19	0 [−0.24; 0.24]
		CG	1.33 (0.37)	19	
	10MWT/DT (m/s)	EG	1.69 (0.24)	19	+0.08 [−0.07; 0.23]
		CG	1.61 (0.22)	19	
	FGA (points)	EG	25.3 (4.6)	19	+1.30 [−1.63; 4.23]
		CG	24.0 (4.3)	19	
	TUGT (s)	EG	11.3 (4.7)	19	−0.5 [−3.37; 2.37]
		CG	11.8 (4.0)	19	

Legend: 10MWT—10-Meter Walk Test; 6MWT—6-Minute Walking Test; ABCS—Activities-specific Balance Confidence Scale; BBS—Berg Balance Scale; CG—Control Group; CI—Confidence Interval; DGI—Dynamic Gait Index; DT—Dual-task; EG—Experimental Group; FES-I—Falls Efficacy Scale-International; FGA—Functional Gait Assessment; LOS—Limit of Stability; Mini-BESTest—Mini Balance Evaluation Systems Test; POMA—Performance Oriented Mobility Assessment; SD—Standard Deviation; ST—Single-task; TUGT—Timed Up and Go Test; UPDRS III—The third part of Unified Parkinson’s Disease Rating Scale. * Mean differences and confidence intervals were calculated by the review authors. ** Mean and standard deviation were estimated from sample size, median, and interquartile range. ^‡^ Indicates comparisons where the control group showed more favorable results than the experimental group. Bolded mean differences (95% CI) indicate statistical significance (CI does not include the null); non-bold values are not statistically significant.

**Table 5:** Primary Outcomes—Post-Intervention Means, Sample Sizes, Mean Differences, and 95% Confidence Intervals from Studies Combining Virtual Reality and Physical Therapy.

Group II: Virtual Reality Training Combined with Physical Therapy					
Study	Outcome	Group	Mean (SD) Post-Intervention	*n*	Mean Difference (95% CI) *
Maranesi et al., 2022 [17]	FES-I (points)	EG	13.3 (1.4)	16	−0.8 [−2.00, 0.40]
		CG	14.1 (1.8)	14	
	Gait Speed (m/s) **	EG	1.8 (0.1)	16	+0.1 [−0.36; 0.56]
		CG	1.7 (0.9)	14	
	POMA (points)	EG	25.9 (0.7)	16	+2.6 [1.70; 3.50]
		CG	23.3 (1.6)	14	
Kashif et al., 2024 [15]	BBS (points)	EG	50.10 (4.90)	20	+4.60 [1.57; 7.63]
		CG	45.50 (4.56)	20	
	ABCS (%)	EG	78.59 (6.39)	20	+7.03
		CG	71.56 (8.09)	20	[2.36, 11.70]
	UPDRS III (points)	EG	17.20 (9.45)	20	−7.25 [−12.15, −2.35]
		CG	24.45 (5.27)	20	

Legend: ABCS—Activities-specific Balance Confidence Scale; BBS—Berg Balance Scale; CG—Control Group; CI—Confidence Interval; EG—Experimental Group; FES-I—Falls Efficacy Scale-International; POMA—Performance Oriented Mobility Assessment; SD—Standard Deviation; UPDRS III—The third part of Unified Parkinson’s Disease Rating Scale. * Mean differences and confidence intervals were calculated by the review authors. ** Test distance or protocol not specified. Non-bold values are not statistically significant.

**Table 6:** Secondary Outcomes—Post-Intervention Means, Sample Sizes, Mean Differences, and Confidence Intervals from Studies Using Virtual Reality Interventions.

Group I: Virtual Reality Training Only					
Study	Outcome	Group	Mean (SD) Post-Intervention	*n*	Mean Difference (95% CI) *
Pazzaglia et al., 2020 [13]	DASH (points)	EG	21.6 (15.1)	25	−3.5 [−12.2, 5.2]
		CG	25.1 (15.8)	26	
	Physical composite-SF-36 (points)	EG	36.8 (9.4)	25	**−9.9 ^‡^** **[−16.57, −3.23]**
		CG	46.7 (13.8)	26	
	Mental composite SF-36 (points)	EG	43.5 (9.2)	25	+4.3 [−1.93, 10.53]
		CG	39.2 (12.6)	26	
Da Silva et al., 2023 [16]	5xSTS (s)	EG	15.70 (4.86)	18	−1.62 [−5.03, 1.79]
		CG	17.32 (5.44)	20	
	PDQ-39 (points)	EG	33.62 (14.63)	18	+2.77 ^‡^ [−7.05, 12.59]
		CG	30.85 (15.14)	20	
Pullia et al., 2023 [14]	FIM (points)	EG	120.57 (5.33)	10	**+21.24** **[3.18, 39.3]**
		CG	99.33 (26.66)	10	
Nuvolini et al., 2025 [19]	MoCA (points)	EG	23.3 (4.2)	19	−0.6 ^‡^ [−3.36, 2.16]
		CG	23.9 (4.2)	19	

Legend: 5xSTS—Five Times Sit-to-Stand Test; CG—Control Group; CI—Confidence Interval; DASH—Disabilities of the Arm, Shoulder and Hand Questionnaire; EG—Experimental Group; FIM—Functional Independence Measure; MoCA—Montreal Cognitive Assessment; PDQ-39—Parkinson’s Disease Questionnaire; SD—Standard Deviation; SF-36—36-Item Short Form Health Survey. * Mean differences and confidence intervals were calculated by the review authors. ^‡^ Indicates comparisons where the control group showed more favorable results than the experimental group. Bolded mean differences (95% CI) indicate statistical significance (CI does not include the null); non-bold values are not statistically significant.

**Table 7:** Secondary Outcomes—Post-Intervention Means, Sample Sizes, Mean Differences, and 95% Confidence Intervals from Studies Combining Virtual Reality and Physical Therapy.

Group II: Virtual Reality Training Combined with Physical Therapy					
Study	Outcome	Group	Mean (SD) Post-Intervention	*n*	Mean Difference (95% CI) *
Maranesi et al., 2022 [17]	BI (points)	EG	94.3 (3.7)	16	**+6.7** **[3.63, 9.77]**
		CG	87.6 (4.5)	14	
	SF-12 (points)	EG	30.1 (0.6)	16	−0.2 ^‡^ [−0.69, 0.29]
		CG	30.3 (0.7)	14	
Kashif et al., 2024 [15]	UPDRS II (points)	EG	15.30 (2.36)	20	**−4.2** **[−6.19, −2.21]**
		CG	19.50 (2.96)	20	

Legend: BI—Barthel Index; CG– Control Group; CI—Confidence Interval; EG—Experimental Group; SD—Standard Deviation; SF-12—12-Item Short Form Health Survey; UPDRS II—The second part of Unified Parkinson’s Disease Rating Scale. * Mean differences and confidence intervals were calculated by the review authors. ^‡^ Indicates comparisons where the control group showed more favorable results than the experimental group. Bolded mean differences (95% CI) indicate statistical significance (CI does not include the null); non-bold values are not statistically significant.

## Supplementary Materials

The following supporting information can be downloaded at: https://www.mdpi.com/article/10.3390/s25154795/s1, Table S1: PRISMA 2020 for Abstracts Checklist; Table S2: PRISMA 2020 for Abstracts Checklist [26].

## Appendix A

*Full Search Strategies*

PubMed:

The search strategy was as follows:

(1) “Parkinson’s Disease [MeSH Terms]”;
(2) “((Parkinson Disease [Title/Abstract]) OR (PD[Title/Abstract])) OR (Parkinsonian [Title/Abstract])”;
(3) “#1 OR #2”;
(4) “(Physical Therapy Modalities [MeSH Terms]) OR (Exercise Therapy [MeSH Terms])”;
(5) “((((((((((((Physical therapy[Title/Abstract]) OR (Physical therapy modalit*[Title/Abstract])) OR (Physical therapy technique*[Title/Abstract])) OR (Physiotherapy[Title/Abstract])) OR (physiotherapy modalit*[Title/Abstract])) OR (physiotherapy technique*[Title/Abstract])) OR (rehabilitation[Title/Abstract])) OR (rehabilitation modalit*[Title/Abstract])) OR (rehabilitation technique*[Title/Abstract])) OR (rehabilitation exercise*[Title/Abstract]))) OR (exercise therap*[Title/Abstract])) OR (remedial exercise*[Title/Abstract])”;
(6) “#4 OR #5”;
(7) “Virtual Reality [MeSH Terms]”;
(8) “(((((((((VR[Title/Abstract]) OR (Immersive virtual reality[Title/Abstract])) OR (Non-immersive virtual reality[Title/Abstract])) OR (Virtual environments[Title/Abstract])) OR (Virtual simulation[Title/Abstract])) OR (3D virtual environment[Title/Abstract])) OR (Immersive technology[Title/Abstract]))) OR (exergam*[Title/Abstract])) OR (serious games[Title/Abstract])”;
(9) “#7 OR #8”;
(10) “#6 AND #10”;
(11) “(Postural Balance [MeSH Terms]) OR (Postural Control [MeSH Terms])”;
(12) “(((((((Postural stability [Title/Abstract]) OR (Dynamic balance [Title/Abstract])) OR (Static balance [Title/Abstract])) OR (Balance ability [Title/Abstract])) OR (Balance performance [Title/Abstract])) OR (Balance control [Title/Abstract])) OR (Balance function [Title/Abstract])) OR (Motor control [Title/Abstract])”;
(13) “#11 OR #12”;
(14) “(Gait [MeSH Terms]) OR (walking [MeSH Terms])”;
(15) “(((((Ambulation [Title/Abstract]) OR (Gait performance [Title/Abstract])) OR (Gait pattern [Title/Abstract])) OR (Gait function [Title/Abstract])) OR (Gait speed [Title/Abstract])) OR (Walking pattern [Title/Abstract])”;
(16) “#14 OR #15”;
(17) “#13 OR #16”;
(18) “#3 AND #10 AND #17”.

Web of Science:

The search strategy was as follows:

(1) “((TS=(Parkinson Disease)) OR TS=(PD)) OR TS=(Parkinsonian)”;
(2) “(((((((((((TS=(Physical therapy)) OR TS=(Physical therapy modalit*)) OR TS=(Physical therapy technique*)) OR TS=(Physiotherapy)) OR TS=(physiotherapy modalit*)) OR TS=(physiotherapy technique*)) OR TS=(rehabilitation)) OR TS=(rehabilitation mo-dalit*)) OR TS=(rehabilitation technique*)) OR TS=(rehabilitation exercise*)) OR TS=(exercise therap*)) OR TS=(remedial exercise*)”;
(3) “(((((((((((TS=(VR)) OR TS=(Immersive virtual reality)) OR TS=(Non-immersive virtual reality)) OR TS=(Virtual environments)) OR TS=(Virtual simulation)) OR TS=(3D virtual environment)) OR TS=(Immersive technology)) OR TS=(exergam*)) OR TS=(serious games)) OR TS=(kinect)) OR TS=(Wii)) OR TS=(X-box)”;
(4) “#2 AND #3”;
(5) “(((((((TS=(Postural stability)) OR TS=(Dynamic balance)) OR TS=(Static balance)) OR TS=(Balance ability)) OR TS=(Balance performance)) OR TS=(Balance control)) OR TS=(Balance function)) OR TS=(Motor control)”;
(6) “((((TS=(Ambulation)) OR TS=(Gait performance)) OR TS=(Gait pattern)) OR TS=(Gait function)) OR TS=(Gait speed)”;
(7) “#5 OR #6”;
(8) “#1 AND #4 AND #7”.
